# 9-Ethyl-3,6-bis­(5-iodo-2-thien­yl)-9*H*-carbazole

**DOI:** 10.1107/S1600536810004484

**Published:** 2010-02-10

**Authors:** Guo-Yi Xu, Wen-Qian Geng, Hong-Ping Zhou

**Affiliations:** a Deparment of Chemistry, Anhui University, Hefei 230039, People’s Republic of China and, Key Laboratory of Functional Inorganic Materials, Chemistry, Hefei 230039, People’s Republic of China

## Abstract

In the title compound, C_22_H_15_I_2_NS_2_, the two thio­phene rings are twisted out of the plane of the central pyrrole ring, making dihedral angles of 32.4 (2)° and 9.8 (2). In the crystal, neighboring mol­ecules are linked into centrosymmetric dimers by pairs of C—H⋯I inter­actions.

## Related literature

For the crystal structures of related carbazole derivatives, see: Yang *et al.* (2005 ▶); Zhou *et al.* (2007 ▶); Zhou *et al.* (2008 ▶); Chen *et al.* (2009 ▶). 
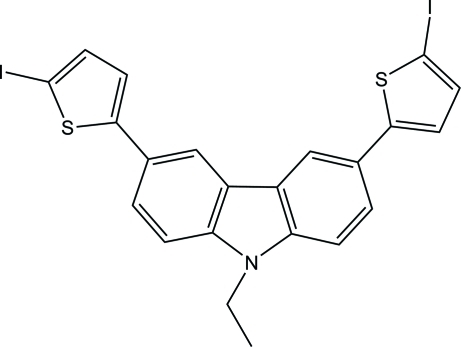


## Experimental

### 

#### Crystal data

C_22_H_15_I_2_NS_2_
*M* *_r_* = 611.29
Monoclinic, 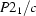

*a* = 10.637 (3) Å
*b* = 7.814 (2) Å
*c* = 26.687 (7) Å
β = 107.313 (18)°
*V* = 2117.7 (10) Å^3^
*Z* = 4
Mo *K*α radiation
μ = 3.17 mm^−1^
*T* = 298 K
0.30 × 0.20 × 0.10 mm

#### Data collection

Bruker SMART CCD area-detector diffractometer
Absorption correction: multi-scan (*SADABS*; Sheldrick, 1996 ▶) *T*_min_ = 0.449, *T*_max_ = 0.742
17471 measured reflections
3738 independent reflections
3065 reflections with *I* > 2σ(*I*)
*R*_int_ = 0.022

#### Refinement

*R*[*F* ^2^ > 2σ(*F* ^2^)] = 0.036
*wR*(*F* ^2^) = 0.151
*S* = 1.16
3738 reflections
245 parameters
H-atom parameters constrained
Δρ_max_ = 1.08 e Å^−3^
Δρ_min_ = −0.79 e Å^−3^

### 

Data collection: *SMART* (Bruker, 2002 ▶); cell refinement: *SAINT* (Bruker, 2002 ▶); data reduction: *SAINT*; program(s) used to solve structure: *SHELXS97* (Sheldrick, 2008 ▶); program(s) used to refine structure: *SHELXL97* (Sheldrick, 2008 ▶); molecular graphics: *SHELXTL* (Sheldrick, 2008 ▶); software used to prepare material for publication: *SHELXTL*.

## Supplementary Material

Crystal structure: contains datablocks I, Global. DOI: 10.1107/S1600536810004484/rk2188sup1.cif

Structure factors: contains datablocks I. DOI: 10.1107/S1600536810004484/rk2188Isup2.hkl

Additional supplementary materials:  crystallographic information; 3D view; checkCIF report

## Figures and Tables

**Table 1:** Hydrogen-bond geometry (Å, °)

*D*—H⋯*A*	*D*—H	H⋯*A*	*D*⋯*A*	*D*—H⋯*A*
C17—H17⋯I1^i^	0.93	3.15	4.040 (9)	161

Symmetry code: (i) 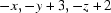.

**Fig. 1.**
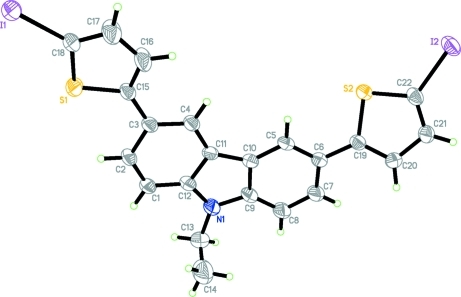
The molecular structure of the title compound withb the atom numbering scheme. Displacement ellipsoids are drawn at the 30% probability level. The H atoms are presented as a small spheres of arbitrary radius.

**Fig. 2.**
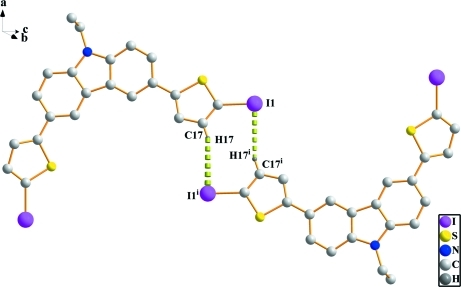
The packing diagram of the title compound. Dashed lines indicate a centrosymmetric dimer C17—H17···I1i. Symmetry code: (i) -x, 3-y, 2-z). H atoms not involved in hydrogen bonds are omitted for clarity.

## supplementary crystallographic information

### Comment

Carbazole - based materials had been investigated for their electrical and optical properties. Especially, introduction of substituents on the 3- and 6-positions of carbazole represents a possible approach for designing carbazole-based photorefractive materials (Yang *et al.*,  2005). The title molecule that we has designed and synthesized is a good intermediate and penetratingly investigated. In the title molecule (Fig.1), the bond lengths and angles show normal values (Chen *et al.*, 2009; Zhou *et al.*, 2007, 2008). Two thiophene rings are twisted out of the plane of the center pyrrole ring and the dihedral angles are 32.4 (2)° and 9.8 (2)°, respectively. In the crystal structure of title compound (Fig.2), the neighboring molecules form a centrosymmetric dimer by C17—H17···I1^i^ (symmetry code: (i) -*x*, 3-*y*, 2-*z*). The neighboring dimers are stacked through weak π···π interaction and the face-to-face distance between two neighboring thiophene rings is 3.57 (4)Å.

### Experimental

Preparation of 9-ethyl-3,6-diiodocarbazole: 9-ethylcarbazole (10 g, 51 mmol) and anhydrous ethanol (150 ml) were added to a three-necked flask equipped with a magnetic stirrer, a reflux condenser and an isobaric dropping funnel. ICl (20 g, 123 mmol)/ethanol (20 ml) was added to the mixture at 353 K. The reaction mixture was refluxed for 2 h, cooled to room temperature and filtered. The grey needle crystals (20.52 g, yield 90%) were obtained and washed with ethanol.

Preparation of 9-ethyl-3,6-di(2-thienyl)carbazole: a 80 ml three-necked round-bottomed flask was charged with  of 9-ethyl-3,6-diiodocarbazole (3.00 g, 6 mmol), 10 ml of DMF, 10 ml of *Et*_3_N and thiophen-2-yl-boronic acid (2.55 g, 20 mmol). A catalytic amount of Pd(O*Ac*)_2_ was added to the stirring solution at 343 K after 9-ethyl-3,6-diiodocarbazole was completely dissolved under nitrogen. The solution was refluxed for 6 h at 403 K. At the end of the reaction was judged by *TLC* analysis. The solution was cooled to room temperature and dissolved in 200 ml CH_2_Cl_2_, then washed with water (3× 200 ml), dried over anhydrous MgSO_4_, brown column crystals were obtained (2.00 g, yield 80% ).

Preparation of 9-ethyl-3,6-di{2-[(5-iodo)thiophene]-yl}carbazole: a 50 ml round-bottomed flask was charged with 9-ethyl-3,6-di(2-thienyl)carbazole (0.13 g, 0.3 mmol) and 9 ml of acetone. A *N*-iodosuccinimide (0.42 g, 1.9 mmol) dissolved in 4 ml acetone and was added to the stirring solution at room temperature after 9-ethyl-3,6-di(2-thienyl)carbazole was completely dissolved. At the end of the reaction was judged by *TLC* analysis after 4 h. The solution was dissolved in 100 ml CH_2_Cl_2_, then washed with water (3× 200 ml), dried over anhydrous MgSO_4_. The organic layer was concentrated in vacuum, the orange suspended solution was decanted out of the flask after 3 ml ethanol was added, green crystals were obtained (0.14 g, yield 54%).

### Refinement

All hydrogen atoms were placed in geometrically idealized positions and constrained to ride on their parent atoms, with C—H = 0.93–0.97Å and *U*_iso_(H) = 1.2–1.5 *U*_eq_(C). At the end of the refinement, the highest peak in the electron–density map was 0.93Å from I2 and the deepest hole was 0.73 Å from I2.

### Crystal data

**Table:**

C_22_H_15_I_2_NS_2_	*F*(000) = 1168
*M**_r_* = 611.29	*D*_x_ = 1.917 Mg m^−^^3^
Monoclinic, *P*2_1_/*c*	Mo *K*α radiation, λ = 0.71069 Å
Hall symbol: -P 2ybc	Cell parameters from 7193 reflections
*a* = 10.637 (3) Å	θ = 2.4–27.4°
*b* = 7.814 (2) Å	µ = 3.17 mm^−^^1^
*c* = 26.687 (7) Å	*T* = 298 K
β = 107.313 (18)°	Prism, green
*V* = 2117.7 (10) Å^3^	0.30 × 0.20 × 0.10 mm
*Z* = 4	

### Data collection

**Table:**

Bruker SMART CCD area-detector diffractometer	3738 independent reflections
Radiation source: fine-focus sealed tube	3065 reflections with *I* > 2σ(*I*)
graphite	*R*_int_ = 0.022
φ– and ω–scans	θ_max_ = 25.0°, θ_min_ = 1.6°
Absorption correction: multi-scan (*SADABS*; Sheldrick, 1996)	*h* = −12→12
*T*_min_ = 0.449, *T*_max_ = 0.742	*k* = −9→9
17471 measured reflections	*l* = −31→30

### Refinement

**Table:**

Refinement on *F*^2^	Primary atom site location: structure-invariant direct methods
Least-squares matrix: full	Secondary atom site location: difference Fourier map
*R*[*F*^2^ > 2σ(*F*^2^)] = 0.036	Hydrogen site location: inferred from neighbouring sites
*wR*(*F*^2^) = 0.151	H-atom parameters constrained
*S* = 1.16	*w* = 1/[σ^2^(*F*_o_^2^) + (0.1*P*)^2^] where *P* = (*F*_o_^2^ + 2*F*_c_^2^)/3
3738 reflections	(Δ/σ)_max_ = 0.002
245 parameters	Δρ_max_ = 1.08 e Å^−^^3^
0 restraints	Δρ_min_ = −0.79 e Å^−^^3^

### Special details

**Table:**

Geometry. All s.u.'s (except the s.u. in the dihedral angle between two l.s. planes) are estimated using the full covariance matrix. The cell s.u.'s are taken into account individually in the estimation of s.u.'s in distances, angles and torsion angles; correlations between s.u.'s in cell parameters are only used when they are defined by crystal symmetry. An approximate (isotropic) treatment of cell s.u.'s is used for estimating s.u.'s involving l.s. planes.
Refinement. Refinement of *F*^2^ against ALL reflections. The weighted *R*-factor *wR* and goodness of fit *S* are based on *F*^2^, conventional *R*-factors *R* are based on *F*, with *F* set to zero for negative *F*^2^. The threshold expression of *F*^2^ > σ(*F*^2^) is used only for calculating *R*-factors(gt) *etc*. and is not relevant to the choice of reflections for refinement. *R*-factors based on *F*^2^ are statistically about twice as large as those based on *F*, and *R*-factors based on ALL data will be even larger.

### Fractional atomic coordinates and isotropic or equivalent isotropic displacement parameters (Å^2^)

**Table:**

	*x*	*y*	*z*	*U*_iso_*/*U*_eq_	
I1	0.25459 (4)	1.46648 (5)	1.073693 (15)	0.0711 (2)	
I2	−0.70485 (4)	0.47106 (5)	0.685869 (19)	0.0818 (2)	
S1	0.28672 (13)	1.0941 (2)	1.02087 (5)	0.0797 (5)	
S2	−0.39715 (12)	0.45973 (15)	0.75996 (5)	0.0589 (3)	
N1	0.2462 (4)	0.3710 (7)	0.87990 (17)	0.0768 (13)	
C1	0.3499 (4)	0.6105 (8)	0.94135 (18)	0.0651 (13)	
H1	0.4337	0.5626	0.9523	0.078*	
C2	0.3241 (4)	0.7652 (8)	0.96071 (18)	0.0631 (12)	
H2	0.3925	0.8228	0.9847	0.076*	
C3	0.1979 (4)	0.8397 (7)	0.94558 (16)	0.0550 (11)	
C4	0.0960 (4)	0.7564 (6)	0.90838 (16)	0.0532 (10)	
H4	0.0125	0.8050	0.8974	0.064*	
C5	−0.0883 (5)	0.4929 (6)	0.81521 (18)	0.0513 (11)	
H5	−0.1440	0.5816	0.8182	0.062*	
C6	−0.1344 (4)	0.3655 (6)	0.77807 (17)	0.0543 (11)	
C7	−0.0498 (5)	0.2311 (7)	0.7748 (2)	0.0721 (14)	
H7	−0.0812	0.1450	0.7502	0.087*	
C8	0.0776 (5)	0.2224 (8)	0.8067 (2)	0.0792 (16)	
H8	0.1326	0.1332	0.8035	0.095*	
C9	0.1219 (5)	0.3487 (7)	0.8434 (2)	0.0674 (13)	
C10	0.0396 (5)	0.4886 (6)	0.84774 (19)	0.0526 (11)	
C11	0.1197 (4)	0.6004 (6)	0.88770 (16)	0.0504 (10)	
C12	0.2465 (5)	0.5279 (7)	0.9049 (2)	0.0612 (13)	
C13	0.3602 (6)	0.2581 (11)	0.8866 (3)	0.101 (2)	
H13A	0.4207	0.2705	0.9217	0.121*	
H13B	0.3318	0.1397	0.8817	0.121*	
C14	0.4210 (8)	0.3038 (11)	0.8499 (3)	0.120 (3)	
H14A	0.4396	0.4243	0.8525	0.180*	
H14B	0.3641	0.2781	0.8154	0.180*	
H14C	0.5018	0.2411	0.8560	0.180*	
C15	0.1743 (5)	1.0054 (6)	0.96736 (18)	0.0521 (11)	
C16	0.0690 (6)	1.1130 (8)	0.9511 (3)	0.0900 (19)	
H16	−0.0044	1.0889	0.9227	0.108*	
C17	0.0809 (6)	1.2599 (9)	0.9803 (3)	0.098 (2)	
H17	0.0165	1.3445	0.9732	0.117*	
C18	0.1926 (4)	1.2712 (6)	1.01974 (17)	0.0570 (11)	
C19	−0.2682 (4)	0.3707 (6)	0.74207 (17)	0.0520 (10)	
C20	−0.3136 (5)	0.3054 (7)	0.69241 (19)	0.0640 (13)	
H20	−0.2592	0.2528	0.6754	0.077*	
C21	−0.4518 (5)	0.3255 (7)	0.6691 (2)	0.0672 (13)	
H21	−0.4972	0.2872	0.6356	0.081*	
C22	−0.5089 (4)	0.4058 (6)	0.7009 (2)	0.0599 (12)	

### Atomic displacement parameters (Å^2^)

**Table:**

	*U*^11^	*U*^22^	*U*^33^	*U*^12^	*U*^13^	*U*^23^
I1	0.0604 (3)	0.0777 (3)	0.0697 (3)	0.00741 (16)	0.0107 (2)	−0.00697 (16)
I2	0.0464 (3)	0.0720 (3)	0.1149 (4)	0.00143 (15)	0.0054 (2)	−0.02046 (19)
S1	0.0533 (8)	0.1005 (10)	0.0672 (8)	0.0256 (8)	−0.0095 (6)	−0.0250 (8)
S2	0.0444 (7)	0.0653 (8)	0.0655 (8)	0.0031 (5)	0.0137 (6)	−0.0110 (5)
N1	0.047 (2)	0.089 (3)	0.086 (3)	0.019 (2)	0.007 (2)	−0.025 (2)
C1	0.041 (2)	0.091 (4)	0.059 (3)	0.017 (3)	0.007 (2)	−0.006 (3)
C2	0.038 (2)	0.093 (4)	0.050 (2)	0.009 (2)	0.0012 (18)	−0.001 (2)
C3	0.041 (2)	0.080 (3)	0.043 (2)	0.007 (2)	0.0110 (18)	0.003 (2)
C4	0.041 (2)	0.071 (3)	0.047 (2)	0.004 (2)	0.0113 (18)	0.005 (2)
C5	0.040 (2)	0.058 (2)	0.059 (3)	0.0080 (19)	0.020 (2)	0.005 (2)
C6	0.044 (2)	0.064 (3)	0.058 (3)	−0.003 (2)	0.0201 (19)	−0.004 (2)
C7	0.053 (3)	0.073 (3)	0.089 (4)	0.001 (3)	0.020 (3)	−0.025 (3)
C8	0.055 (3)	0.081 (4)	0.097 (4)	0.018 (3)	0.016 (3)	−0.027 (3)
C9	0.042 (2)	0.080 (3)	0.077 (3)	0.015 (3)	0.012 (2)	−0.008 (3)
C10	0.042 (3)	0.060 (2)	0.057 (3)	0.005 (2)	0.018 (2)	−0.001 (2)
C11	0.040 (2)	0.068 (3)	0.045 (2)	0.010 (2)	0.0170 (18)	0.004 (2)
C12	0.040 (3)	0.081 (3)	0.062 (3)	0.011 (2)	0.013 (2)	−0.004 (2)
C13	0.070 (4)	0.129 (6)	0.107 (5)	0.007 (4)	0.032 (4)	−0.041 (5)
C14	0.100 (5)	0.111 (6)	0.141 (7)	−0.020 (5)	0.023 (5)	−0.026 (5)
C15	0.038 (2)	0.068 (3)	0.045 (2)	0.004 (2)	0.0054 (19)	0.0058 (19)
C16	0.060 (3)	0.079 (4)	0.101 (4)	0.018 (3)	−0.023 (3)	−0.016 (3)
C17	0.070 (4)	0.077 (4)	0.116 (5)	0.023 (3)	−0.019 (3)	−0.015 (4)
C18	0.044 (2)	0.065 (3)	0.056 (3)	0.004 (2)	0.0054 (19)	0.004 (2)
C19	0.043 (2)	0.051 (2)	0.063 (3)	−0.003 (2)	0.0174 (19)	−0.005 (2)
C20	0.055 (3)	0.076 (3)	0.064 (3)	−0.004 (3)	0.022 (2)	−0.017 (2)
C21	0.053 (3)	0.069 (3)	0.074 (3)	−0.008 (3)	0.011 (2)	−0.025 (3)
C22	0.044 (2)	0.050 (2)	0.080 (3)	−0.005 (2)	0.010 (2)	−0.008 (2)

### Geometric parameters (Å, °)

**Table:**

I1—C18	2.066 (5)	C7—C8	1.371 (7)
I2—C22	2.066 (5)	C7—H7	0.9300
S1—C18	1.703 (5)	C8—C9	1.372 (7)
S1—C15	1.713 (5)	C8—H8	0.9300
S2—C22	1.720 (5)	C9—C10	1.427 (7)
S2—C19	1.727 (4)	C10—C11	1.444 (7)
N1—C12	1.395 (7)	C11—C12	1.408 (6)
N1—C9	1.400 (6)	C13—C14	1.373 (9)
N1—C13	1.466 (8)	C13—H13A	0.9700
C1—C2	1.374 (8)	C13—H13B	0.9700
C1—C12	1.392 (7)	C14—H14A	0.9600
C1—H1	0.9300	C14—H14B	0.9600
C2—C3	1.407 (6)	C14—H14C	0.9600
C2—H2	0.9300	C15—C16	1.364 (7)
C3—C4	1.394 (6)	C16—C17	1.373 (9)
C3—C15	1.471 (7)	C16—H16	0.9300
C4—C11	1.392 (7)	C17—C18	1.336 (7)
C4—H4	0.9300	C17—H17	0.9300
C5—C10	1.380 (7)	C19—C20	1.367 (6)
C5—C6	1.387 (6)	C20—C21	1.425 (7)
C5—H5	0.9300	C20—H20	0.9300
C6—C7	1.403 (7)	C21—C22	1.338 (7)
C6—C19	1.462 (6)	C21—H21	0.9300
			
C18—S1—C15	93.1 (2)	C1—C12—C11	121.5 (5)
C22—S2—C19	92.1 (2)	N1—C12—C11	109.5 (4)
C12—N1—C9	108.0 (4)	C14—C13—N1	107.7 (7)
C12—N1—C13	125.9 (5)	C14—C13—H13A	110.2
C9—N1—C13	125.8 (5)	N1—C13—H13A	110.2
C2—C1—C12	117.8 (4)	C14—C13—H13B	110.2
C2—C1—H1	121.1	N1—C13—H13B	110.2
C12—C1—H1	121.1	H13A—C13—H13B	108.5
C1—C2—C3	122.5 (5)	C13—C14—H14A	109.5
C1—C2—H2	118.8	C13—C14—H14B	109.5
C3—C2—H2	118.8	H14A—C14—H14B	109.5
C4—C3—C2	119.0 (5)	C13—C14—H14C	109.5
C4—C3—C15	120.3 (4)	H14A—C14—H14C	109.5
C2—C3—C15	120.8 (4)	H14B—C14—H14C	109.5
C11—C4—C3	119.8 (4)	C16—C15—C3	129.6 (5)
C11—C4—H4	120.1	C16—C15—S1	108.6 (4)
C3—C4—H4	120.1	C3—C15—S1	121.8 (3)
C10—C5—C6	120.3 (4)	C15—C16—C17	114.0 (5)
C10—C5—H5	119.9	C15—C16—H16	123.0
C6—C5—H5	119.9	C17—C16—H16	123.0
C5—C6—C7	119.0 (4)	C18—C17—C16	114.4 (5)
C5—C6—C19	121.2 (4)	C18—C17—H17	122.8
C7—C6—C19	119.8 (4)	C16—C17—H17	122.8
C8—C7—C6	122.2 (5)	C17—C18—S1	109.9 (4)
C8—C7—H7	118.9	C17—C18—I1	128.5 (4)
C6—C7—H7	118.9	S1—C18—I1	121.6 (2)
C7—C8—C9	118.2 (5)	C20—C19—C6	128.3 (4)
C7—C8—H8	120.9	C20—C19—S2	109.8 (3)
C9—C8—H8	120.9	C6—C19—S2	121.9 (3)
C8—C9—N1	129.7 (5)	C19—C20—C21	113.7 (4)
C8—C9—C10	121.4 (5)	C19—C20—H20	123.1
N1—C9—C10	108.9 (4)	C21—C20—H20	123.1
C5—C10—C9	118.8 (4)	C22—C21—C20	112.2 (4)
C5—C10—C11	134.8 (4)	C22—C21—H21	123.9
C9—C10—C11	106.4 (4)	C20—C21—H21	123.9
C4—C11—C12	119.5 (4)	C21—C22—S2	112.1 (4)
C4—C11—C10	133.3 (4)	C21—C22—I2	127.9 (4)
C12—C11—C10	107.1 (4)	S2—C22—I2	120.0 (3)
C1—C12—N1	129.1 (4)		
			
C12—C1—C2—C3	1.0 (8)	C13—N1—C12—C11	178.7 (6)
C1—C2—C3—C4	−2.0 (8)	C4—C11—C12—C1	−1.4 (7)
C1—C2—C3—C15	179.8 (5)	C10—C11—C12—C1	176.1 (5)
C2—C3—C4—C11	1.3 (7)	C4—C11—C12—N1	178.6 (4)
C15—C3—C4—C11	179.5 (4)	C10—C11—C12—N1	−3.9 (6)
C10—C5—C6—C7	1.2 (7)	C12—N1—C13—C14	−90.0 (8)
C10—C5—C6—C19	−178.0 (4)	C9—N1—C13—C14	83.0 (9)
C5—C6—C7—C8	−0.9 (8)	C4—C3—C15—C16	−12.0 (8)
C19—C6—C7—C8	178.3 (5)	C2—C3—C15—C16	166.2 (6)
C6—C7—C8—C9	1.2 (9)	C4—C3—C15—S1	168.4 (4)
C7—C8—C9—N1	−179.2 (6)	C2—C3—C15—S1	−13.4 (6)
C7—C8—C9—C10	−1.7 (9)	C18—S1—C15—C16	−0.7 (5)
C12—N1—C9—C8	174.0 (6)	C18—S1—C15—C3	179.0 (4)
C13—N1—C9—C8	0.0 (11)	C3—C15—C16—C17	−178.9 (6)
C12—N1—C9—C10	−3.7 (6)	S1—C15—C16—C17	0.8 (8)
C13—N1—C9—C10	−177.7 (6)	C15—C16—C17—C18	−0.5 (10)
C6—C5—C10—C9	−1.8 (7)	C16—C17—C18—S1	0.0 (8)
C6—C5—C10—C11	176.5 (5)	C16—C17—C18—I1	179.7 (5)
C8—C9—C10—C5	2.1 (8)	C15—S1—C18—C17	0.4 (5)
N1—C9—C10—C5	180.0 (5)	C15—S1—C18—I1	−179.4 (3)
C8—C9—C10—C11	−176.6 (5)	C5—C6—C19—C20	151.0 (5)
N1—C9—C10—C11	1.3 (6)	C7—C6—C19—C20	−28.1 (8)
C3—C4—C11—C12	0.4 (7)	C5—C6—C19—S2	−31.6 (6)
C3—C4—C11—C10	−176.4 (5)	C7—C6—C19—S2	149.2 (4)
C5—C10—C11—C4	0.2 (9)	C22—S2—C19—C20	0.6 (4)
C9—C10—C11—C4	178.5 (5)	C22—S2—C19—C6	−177.3 (4)
C5—C10—C11—C12	−176.8 (5)	C6—C19—C20—C21	177.0 (5)
C9—C10—C11—C12	1.5 (5)	S2—C19—C20—C21	−0.6 (6)
C2—C1—C12—N1	−179.3 (5)	C19—C20—C21—C22	0.4 (7)
C2—C1—C12—C11	0.7 (8)	C20—C21—C22—S2	0.1 (6)
C9—N1—C12—C1	−175.2 (6)	C20—C21—C22—I2	178.7 (4)
C13—N1—C12—C1	−1.2 (10)	C19—S2—C22—C21	−0.4 (4)
C9—N1—C12—C11	4.7 (7)	C19—S2—C22—I2	−179.1 (3)

### Hydrogen-bond geometry (Å, °)

**Table:**

*D*—H···*A*	*D*—H	H···*A*	*D*···*A*	*D*—H···*A*
C17—H17···I1^i^	0.93	3.15	4.040 (9)	161

Symmetry codes: (i) −*x*, −*y*+3, −*z*+2.

## References

[bb1] Bruker (2002). *SMART*, *SAINT* Bruker AXS Inc., Madison, Wisconsin, USA.

[bb2] Chen, L., Cheng, W., Song, G.-L. & Zhu, H.-J. (2009). *Acta Cryst.* E**65**, o574. 10.1107/S1600536809005583 PMC2968689 21582229

[bb3] Sheldrick, G. M. (1996). *SADABS* University of Goumlttingen, Germany.

[bb4] Sheldrick, G. M. (2008). *Acta Cryst.* A**64**, 112–122. 10.1107/S0108767307043930 18156677

[bb5] Yang, J. X., Tao, X. T., Yuan, C. X., Yan, Y. X., Wang, L., Liu, Zh., Ren, Y. & Jiang, M. H. (2005). *J. Am. Chem. Soc.* **127**, 3278–3279. 10.1021/ja043510s 15755135

[bb6] Zhou, H. P., Lv, L. F., Wang, P. & Hu, R. T. (2008). *Acta Cryst.* E**64**, o1075. 10.1107/S1600536808013937 PMC2961443 21202593

[bb7] Zhou, H. P., Wang, P., Hu, Z. J., Li, L., Chen, J. J., Cui, Y., Tian, Y. P., Wu, J. Y., Yang, J. X., Tao, X. T. & Jiang, M. H. (2007). *Eur. J. Inorg. Chem.* **13**, 1854–1866.

